# On the Preparation for and After-Treatment of Abdominal Operations

**Published:** 1898-12-31

**Authors:** Thomas Carwardine

**Affiliations:** Assistant Surgeon, Bristol Royal Infirmary.


### Hospital Clinics and Medical Progress.

### ON THE PREPARATION FOR AND AFTER-TREATMENT OF ABDOMINAL OPERATIONS.

By Thomas Carwardine, M.S.Lond., F.R.C.S.Eng., Assistant Surgeon, Bristol Royal Infirmary.

(*Continued from page* 210)

When the operation is completed, the sponge nurse counts the sponges and forceps whilst the head nurse hands the dressings, the anæsthetic nurse being at hand in case of vomiting. The sponge nurse is then free to adjust the patient's bed. The pillow should be removed, and a towel placed for the head to rest upon. Another towel is subsequently placed upon the patient's chest. A receiver is handy in case of vomiting. A cradle is placed over the abdomen to support the outer bed-clothes, and a protected hot-water bottle near the feet. There should be two blankets immediately over the patient, which should overlap each other at the abdomen to enable inspection to occur without much disturbance.

**After Treatment.**—First day: The patient should be made comfortable, and afterwards disturbed or worried as little as possible. The temperature, pulse, and respiration must be regularly recorded day and night. An increasingly rapid and feeble pulse on the day of operation is a serious indication either of shock or hæmorrhage. Any pulse exceeding 110 should be immediately reported to the surgeon. Pallor with rapid shallow sighing respirations are signs of shock or hæmorrhage. A subnormal temperature indicates shock, and must be treated by warmth to the body and stimulating enemata until it becomes normal. Rapidity of respiration increases with the onset of pulmonary congestion or pneumonia. A tube should be passed into the rectum every six hours, and be left in for a quarter or half an hour to release flatus. Morphia should always be avoided if possible, and the patient encouraged to bear a little inconvenience for a short time. Ice should not be given. Thirst may be relieved by the mouth a few hours after all vomiting has ceased, otherwise by nutrient enemata or a single copious enema of soap and water. The familiar cup of tea is often the best drink to start with, and it is a pleasure to see the patient enjoy it under the circumstances. To refuse drink in small quantities after vomiting is over is unnecessary, and therefore cruel when thirst is great. Nutrient enemata may be given as soon as the operation is over, and continued as long as necessary, washing out the bowel every 36 hours. They must not exceed four ounces. Hot toast water, barley water, or a little thin, wholesome beef-tea may be given in the evening.

Second day: A slight rise of temperature is a good rather than a bad sign. High temperature may indicate the onset of pneumonia or a septic infection of the wound. If peritonitis is likely to ensue the aspect of the patient will be changed to a pinched, drawn appearance even at this early stage. The patient may ask to be relieved by the catheter 24 hours after operation; it will not be required before, and every care must be taken in passing it. Some peptonized cocoa, oatmeal gruel, or any of the concentrated meat essences may be given, or suitable varieties of infants' foods.

Third day: Peritonitis will usually manifest itself on the morning of the third day if it occur at all. The pulse suddenly runs up to 120 or more, and becomes small. A few hours later the temperature usually, but not always, rises. The belly becomes distended, hard, and board-like; the face is pinched, and hiccough and vomiting may supervene. Alarm may at this time be felt owing to distension of the abdomen with localised tenderness at the lower part, which, however, may not be due to diffuse peritonitis. It is very frequent after morphia has been given on the first night. A copious soap-and-water enema, containing half an ounce of turpentine, will do wonders in these cases, and may be repeated until flatus is freely expelled and the patient is made comfortable. This may be assisted by the passage of the rectum tube. Sulphate of magnesia suitably covered may be given by the mouth with advantage. If the tympanites increases or persists, hypodermic injections of four minims of liquor strychnine hydrochloratis and one-fiftieth of a grain of digitalin should be given. This is, indeed, the best treatment to pursue whenever there is fear of peritonitis, or when it is actually present.

If all is satisfactory the patient may have some bread and butter, tapioca, sago, arrowroot, custard, and tea in moderation,

Fourth day onwards: The patient is usually out of danger as far as immediate complications are concerned. The patient will now be rendered more comfortable by giving a simple enema.

Seventh day: The wound should now be redressed. Stitches should not be removed. The redressing should be done with all the precautions as for operation, macintoshes being placed above and below the wound, and the hands and instruments purified.

Tenth day: Superficial stitches may be removed if the abdomen has been sewn up in layers, but there should be no hurry to remove stitches. The abdomen may afterwards be supported by a broad piece of strapping cut out in the centre like a grating.

Twenty-first day: If the abdomen has been sewn up by sutures transfixing all the tissues, they should not be removed for three weeks. The patient may then get up with the abdomen supported by a broad piece of strapping or flannel binder for support. The patient should return in a year's time for inspection of the scar, and should be told to seek advice at once if she should get any pain, constipation, or vomiting.

The above rules for guidance may be modified to suit the individuality of the surgeon, but they may serve as a reliable and standard guide to those who are more or less responsible for preparatory and after treatment. They are based on the writer's experience of those measures which have proved most satisfactory, and they may accordingly prove useful to others also.

